# Planned home compared with planned hospital births: mode of delivery and Perinatal mortality rates, an observational study

**DOI:** 10.1186/s12884-017-1348-y

**Published:** 2017-06-08

**Authors:** Jacoba van der Kooy, Erwin Birnie, Semiha Denktas, Eric A.P Steegers, Gouke J. Bonsel

**Affiliations:** 1 Department of Obstetrics and Gynecology, Division of Obstetrics & Prenatal Medicine, Room Hs-408, Erasmus MC, PO Box 2040, 3000 CA Rotterdam, The Netherlands; 2 Institute of Health Policy and Management, Erasmus University Rotterdam, PO Box 1738, 3000 DR Rotterdam, The Netherlands; 3 University of Applied Sciences, Midwifery Academy Rotterdam (Verloskunde Academie Rotterdam), Dr. Molewaterplein 40, 3015 GD Rotterdam, The Netherlands; 4 Academic Collaboration Mother and Child Care, Wilhelmina Child Hospital, University Medical Center Utrecht, Postbus 85090, 3508 AB Utrecht, The Netherlands

**Keywords:** Intervention, Mortality, Perinatal care

## Abstract

**Background:**

To compare the mode of delivery between planned home versus planned hospital births and to determine if differences in intervention rates could be interpreted as over- or undertreatment.

**Methods:**

Intervention and perinatal mortality rates were obtained for 679,952 low-risk women from the Dutch Perinatal Registry (2000–2007). Intervention was defined as operative vaginal delivery and/or caesarean section. Perinatal mortality was defined as the intrapartum and early neonatal mortality rate up to 7 days postpartum.

Besides adjustment for maternal and care factors, we included for additional casemix adjustment: presence of congenital abnormality, small for gestational age, preterm birth, or low Apgar score. The techniques used were nested multiple stepwise logistic regression, and stratified analysis for separate risk groups. An intention-to-treat like analysis was performed.

**Results:**

The intervention rate was lower in planned home compared to planned hospital births (10.9% 95% CI 10.8–11.0 vs. 13.8% 95% CI 13.6–13.9). Intended place of birth had significant impact on the likelihood to intervene after adjustment (planned homebirth (OR 0.77 95% CI. 0.75–0.78)).

The mortality rate was lower in planned home births (0.15% vs. 0.18%). After adjustment, the interaction term home- intervention was significant (OR1.51 95% CI 1.25–1.84). In risk groups, a higher perinatal mortality rate was observed in planned home births.

**Conclusions:**

The potential presence of over- or under treatment as expressed by adjusted perinatal mortality differs per risk group. In planned home births especially multiparous women showed universally lower intervention rates. However, the benefit of substantially fewer interventions in the planned home group seems to be counterbalanced by substantially increased mortality if intervention occurs.

## Background

The challenge of obstetric care is to optimize maternal and child health outcomes and the mother’s experience of childbirth with the least possible interventions in the normal process [1]. This challenge has led to a wide debate in recent years about relative benefits and risks of birth in different settings and the associated risk of medical interventions [2–12].

In the Netherlands, approximately 50% of pregnant women start their delivery in primary care under the supervision of a community midwife. Community midwives are independent health care professionals working either solely or in group practices [13] who provide care for low risk and medium risk pregnant women according to Dutch guidelines [14]. Only low risk women can choose their birthplace: at home or in the hospital, both supervised by the community midwife only.

The debate on different birth settings in the Netherland has intensified since the national perinatal mortality rate showed to be one of the highest in Europe, although the difference in perinatal mortality mainly occurs in preterm births [15, 16]. While the proportion of home birth deliveries in the Netherlands has steadily decreased to 17% of all births [17], several high income countries consider the reintroduction of home births [18–20]. This is based on claims of equal safety at lower intervention rates compared to hospital births where overtreatment might be present [20, 21]. Furthermore it is based on the stated reduction of maternal-fetal morbidity and suggested psychosocial advantages for the mother [2, 6, 7, 9–11]. These benefits may be counterbalanced by the disadvantages associated with delayed treatment or even undertreatment in planned home births leading to an increased risk of perinatal mortality, morbidity and long term adverse effects [20, 22, 23]. Studies addressing the benefits and disadvantages of home birth can be challenged due to their observational study design with insufficient casemix adjustment for interventions and outcomes, and exclusion of women from the analysis, which according to the delivery guidelines should have been referred prior to delivery [2, 6–11]. This paper compares the intervention rates between planned home and planned hospital births, and determines whether these can be interpreted as over- or undertreatment by comparing adjusted perinatal mortality rates.

## Methods

### Data

The Netherlands Perinatal Registry (PRN) contains population-based information of 96% of all pregnancies in The Netherlands. Source data are collected by 95% of midwives, 99% of gynecologists and 68% of pediatricians (including 100% of Neonatal Intensive Care Unit pediatricians) [15, 24]. (See https://www.perined.nl for details.) The PRN does not include long term child outcomes. Detailed information on risk factors is only partially available in the PRN registry.

Included were the records of all singleton pregnant women (693,592 women) who at the onset of labor (spontaneous contractions or spontaneous rupture of membranes) were supervised by community midwives between 2000 and 2007.

Excluded were 13,384 women with so called ‘medium risk’, e.g. women with a history of postpartum hemorrhage or obesity, since Dutch guidelines prescribe a hospital delivery with their midwife-led delivery (no choice of planned home birth). Secondly, 256 incomplete data records were excluded. The remaining 679,952 women were categorized according to intended place of birth (home/hospital/unknown (place was undecided/not recorded)).

### Determinants

Maternal determinants were parity (nulliparous/multiparous), age, ethnicity (Western/non-Western; based on a more refined classification in the registry), and living in a deprived neighbourhood (yes/no, based on 4-digit zip-codes and a public list of zip-code based deprived, neighborhoods issued by the Dutch government) [25]. Health care related determinants were time of birth (day 8.00–18.00, night 18.00–8.00), day of birth (week day, weekend) and receiving an intervention (yes/no).

### Outcome measures

Two primary outcomes were defined. First, receiving an intrapartum intervention during delivery (including operative vaginal delivery and/or secondary caesarean section). Second, perinatal mortality, which was defined as the intrapartum and early neonatal mortality up to 7 days postpartum.

### Casemix adjustment

Casemix of any defined group of women was primarily represented by the prevalence of Big4 conditions (see below) selected as most important risk mediators. The presence of any of the four conditions is known to precede perinatal mortality in 85% of cases (PRN dataset, years 2000–2007, 1.25 million records) [26]. These four child conditions are; congenital abnormalities (list defined), intrauterine growth restriction (SGA, birthweight below the 10th percentile for gestational age, gender and parity specific), preterm birth (<37th week of gestation) or low Apgar score (<7, measured 5 min after birth) [26]. We refer to these four conditions as the Big4. In the current analysis Big4 represent an objective estimate of the risk load at birth and therefore it is used for casemix adjustment in this context. In a system with optimal risk selection Big4 conditions should not occur in the low risk population giving birth under the supervision of a community midwife. However, since risk selection is not optimal in the Dutch obstetric care system Big4 conditions are still present in this group.

Casemix adjustment is different for the intervention outcome (an intrapartum measurement) and mortality outcome (a postpartum measurement). When comparing mortality rates Big4 casemix adjustment is used. However, when comparing intervention rates, the intervention precedes the outcome low Apgar score. Low Apgar should therefore be excluded from the Big4. This is referred as Big3 adjustment.

### Statistical analysis

Firstly, we compared characteristics of the population by intended place of birth using Student’s t-tests for continuous variables with normal distributions and chi-square tests for nominal or ordinal variables (Table 1). Secondly, the intervention rate was compared between planned home versus planned hospital births, after Big3 adjustment. The planned place of birth is routinely asked by the midwife at 30 weeks of gestation. An intention-to-treat-like analysis approach was used [27]. It is called intention-to-treat-like since intention-to-treat analysis is mainly used in RCT’s. The intention-to-treat-like analysis approach implies that all women who were able to plan a home or hospital birth were included (so women with so called ‘medium risk’ were excluded), independent from later referral during labor (denominator *n* = 679,952). For the statistical analysis we selected a nested multiple stepwise logistic regression (stepwise analysis; inclusion *p* < 0.05; exclusion *p* > 0.10). Model 1 gives the crude risk ratios. Model 2 gives the adjusted odds ratios, including maternal, child (casemix) and health care related determinants. Thirdly, we compared the perinatal mortality rates after Big4 adjustment using an intention-to-treat-like approach. For this analysis a nested multiple stepwise regression model (stepwise analysis; inclusion *p* < 0.05; exclusion *p* > 0.10) was used (model 1). Additionally we added receiving an intervention (yes/no) and its interaction with intended place of birth as an explaining determinant (model 2) [28].

**Table 1:** Characteristics and outcome of women in primary care at the onset of labour; intention-to-treat-like approach^a^

Variable	Planned home birth		Planned hospital birth		Planned place unknown	
	n	%	n	%	n	%
	402,912	59%	219,105	32%	57,935	9%
Parity**						
Primiparous	171,986	42,7%	104,249	47,6%	26,254	45,3%
Multiparous (REF)	230,926	57,3%	114,856	52,4%	31,681	54,7%
Maternal Age**						
< 19 years	4036	1,0%	6713	3,1%	1190	2,1%
20–25 years	34,661	8,6%	32,617	14,9%	6823	11,8%
25–34 years (REF)	296,128	73,5%	142,597	65,1%	39,526	68,2%
> 35 years	68,087	16,9%	37,178	17,0%	10,396	17,9%
Ethnic background**						
Dutch (REF)	370,647	92,0%	153,572	70,1%	46,966	81,1%
Non Dutch	32,265	8,0%	65,533	29,9%	10,969	18,9%
Neighbourhood**						
Privileged neighbourhood (REF)	388,089	96,3%	196,659	89,8%	53,823	92,9%
Underprivileged neighbourhood	14,823	3,7%	22,446	10,2%	4112	7,1%
Gestational Age**						
< 34wk	2396	0,6%	1658	0,8%	567	1,0%
35-36wk	6510	1,6%	4064	1,9%	1206	2,1%
37wk	15,203	3,8%	9603	4,4%	2497	4,3%
38-41wk (REF)	372,787	92,5%	200,872	91,7%	52,899	91,3%
> 41 wk	6016	1,5%	2908	1,3%	766	1,3%
Big4**						
SGA	28,029	7,0%	18,288	8,3%	4364	7,5%
Prematurity	8056	2,0%	5194	2,4%	1583	2,7%
Low apgar	1642	0,4%	1171	0,5%	290	0,5%
Congenital abnomality	4711	1,2%	2826	1,3%	759	1,3%
Combination Big4	1895	0,5%	1326	0,6%	373	0,6%
No Big4	358,579	89,0%	190,300	86,9%	50,566	87,3%
Time of delivery**						
Day 8.00–18.00 (REF)	167,345	41,5%	96,033	43,8%	24,674	42,6%
Night 18.00–8.00	235,567	58,5%	123,072	56,2%	33,261	57,4%
Day of delivery**						
Weekend	109,761	27,2%	59,976	27,4%	15,553	26,8%
Week day (REF)	293,151	72,8%	159,129	72,6%	42,382	73,2%
Interventions**						
Vacuum extraction/forceps	32,481	8,1%	20,404	9,3%	4630	8,0%
Section cesarean	11,285	2,8%	9731	4,4%	2412	4,2%
No vacuum/forceps or section cesarean	359,146	89,1%	188,970	86,2%	50,893	87,8%
Intrapartum & early neonatal death (7 days)**						
No	402,266	99,8%	218,672	99,8%	57,826	99,8%
Yes	594	0,15%	403	0,18%	102	0,18%

*REF* reference group

^a^Totals may not add up to 100 because of rounding error

** *p* < 0,001 (home vs hospital vs unknown planned place of birth)

In both regression analyses hospital birth was set as reference. All stepwise analyses were repeated with a forward and backward approach. Results of the two approaches were similar unless stated otherwise. For the regression analysis risk factor coefficients were only shown if *p* < 0.05.

Fourthly, the presence of over- and undertreatment for predefined risk groups was assessed by stratifying the women into five predefined risk groups (noBig3, SGA, preterm birth, congenital anomaly, combination Big3) and parity (nulliparous/multiparous), altogether 10 groups. A comparison of unadjusted perinatal mortality rates was made by planned place of birth (home/hospital) and by receiving an intervention (yes/no). The presence of over- or undertreatment was assessed within each group separately by comparing the intervention and perinatal mortality rate. In general undertreatment was suspected if substantial lower intervention rates were present in planned home births compared to planned hospital births, while simultaneously the perinatal mortality rate was higher in planned home births. Overtreatment was suspected when substantial lower intervention rates were present in planned home births compared to planned hospital births, while the mortality rate was lower or equal in planned home births.

Technically, the difference in the intervention rate and the difference in the perinatal mortality rate were both expressed as risk ratios with hospital set as reference (dividing the rate in the planned home birth by rate in the planned hospital birth).

**Fig. 1:**
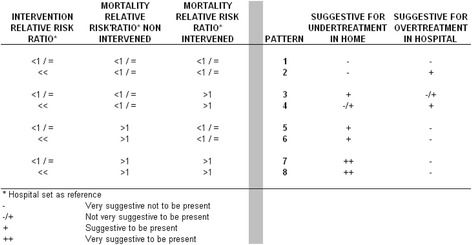
Differences in intervention rate and mortality rate between planned home and hospital births classified into eight patterns

Eight different combinations of the intervention risk ratio and the perinatal mortality ratio were developed, which allowed for the interpretation of over- or undertreatment (Fig. 1). These eight patterns describe the relationship between (1) the risk ratio of an intervention, (2), the risk ratio of perinatal mortality in the intervened group, and (3) the risk ratio of perinatal mortality in the non-intervened group. Risk ratios larger than 1 were bolded.

The risk ratio of an intervention was subdivided into: I. A risk ratio slightly lower or equal to 1.0, indicated as **(<1/=)**, representing a slightly lower tendency to intervene in the planned home birth; II. A risk ratio less than 1.0 (typically in the range 0.3–0.6) indicated as **(<<)**, representing a considerable lower tendency to intervene in home deliveries.

The risk ratio of perinatal mortality was subdivided into: I. a risk ratio slightly lower or equal to 1.0, indicated as **(<1/=)**, representing about equal or a slightly lower perinatal mortality in the planned home birth; II. a risk ratio more than 1.0, indicated as (**>1**), representing a considerable higher mortality in home deliveries.

In Fig.1 patterns 2 and 4 were suggestive for overtreatment, patterns 3, 5 and 6 were suggestive for undertreatment, patterns 7 and 8 were very suggestive for overtreatment, and for pattern 1 over- or undertreatment was not likely to be present. Note that the interpretation of pattern 1 and 2 rest on the assumption that residual confounding might still be present, leading to a favorable casemix in home deliveries.

## Results

Table 1 describes the baseline characteristics of the intention-to-treat-like population (*n* = 679,952).

In the population who started birth under supervision of the community midwife about 59% of women planned a home birth, about 32% planned a hospital birth and 9% planned place of birth was unknown. Compared to women who planned birth in the hospital women with planned home birth were more likely to be multiparous, 25 years or older, of Dutch origin and living in a non-deprived neighbourhood. The prevalence of Big4 conditions was lower in planned home births (11.0%) compared to planned hospital births (13.1%) and planned place unknown (12.7%) (*p* < 0.001).

Interventions were less prevalent in planned home births (10.9%) compared to planned hospital births (13.7%) and to planned place unknown (12.2%) (*p* < 0.001). Intrapartum and neonatal mortality was 0.15% for planned home births, 0.18% for planned hospital births and 0.18% for planned place unknown (*p* < 0.001).

### Intervention rates

The crude intervention risk ratio was significantly lower for women who planned home birth (RR 0.76, [95% CI 0.75–0.78, *p* < 0.001]) compared to those who planned a hospital birth (Table 2, Model 1). All maternal and child risk factors (except the presence of SGA), showed significant differences in RR in agreement with the expected direction.

**Table 2:** Intervention (operative vaginal delivery and caesarean section) in women who are in primary care at the onset of labour

	TOTAL(n)	IV (N)	%	*p*	Model 1			*p*	Model 2			*p*
					Crude RR		95% CI		Adj OR		95% CI	
Intended place of birth				**				**				**
Home	402,912	43,766	0,109		0,76	0,75	0,78		0,77	0,75	0,78	
Hospital (REF)	219,105	30,135	0,138		1				1			
Unknown	57,935	7042	0,122		0,87	0,84	0,89		0,86	0,84	0,89	
Parity				**				**				**
Primiparous	302,489	70,334	0,233		10,49	10,27	10,71		11,90	11,64	12,16	
Multiparous (REF)	377,463	10,609	0,028		1				1			
Maternal Age				**				**				**
< 19 years	11,939	1295	0,108		0,86	0,81	0,91		0,40	0,37	0,42	
20–25 years	74,101	8737	0,118		0,94	0,92	0,96		0,58	0,57	0,60	
25–34 years (REF)	478,251	59,536	0,124		1				1			
> 35 years	115,661	11,375	0,098		0,77	0,75	0,78		1,36	1,33	1,40	
Ethnic background				**				**				**
Dutch (REF)	571,185	69,983	0,123		1				1			
Non Dutch	108,767	10,960	0,101		0,80	0,79	0,82		0,96	0,94	0,99	
Neighbourhood				**				**				**
Privileged neighbourhood (REF)	638,571	76,646	0,120		1				1			
Underprivileged neighbourhood	41,381	4297	0,104		0,85	0,82	0,88		0,90	0,87	0,94	
Gestational Age				**				**				**
< 34wk	4621	873	0,189		1,90	1,76	2,04		1,02	0,94	1,10	
35-36wk	11,780	1926	0,163		1,48	1,41	1,55		0,96	0,91	1,01	
37wk	27,303	2701	0,099		0,83	0,80	0,86		0,63	0,61	0,66	
38-41wk (REF)	626,558	73,286	0,117		1				1			
> 41 wk	9690	2157	0,223		2,16	2,06	2,27		2,11	2,00	2,22	
Big3				**				**				**
SGA	50,681	5169	0,102		0,83	0,81	0,85		0,85	0,82	0,87	
Prematurity	16,401	2799	0,350									
Congenital abnomality	3594	904	0,252		1,86	1,76	1,97		1,77	1,67	1,88	
Combination Big3	2443	507	0,140		2,64	2,44	2,85		1,66	1,49	1,85	
No Big3	606,833	71,564	0,118		1				1			
Time of delivery								**				**
Day 8.00–18.00 (REF)	288,052	38,414	0,133		1				1			
Night 18.00–8.00	391,900	42,529	0,109		0,79	0,78	0,80		0,86	0,84	0,87	
Day of delivery								**				
Week day (REF)	494,662	58,785	0,119		1				nie			
Weekend	185,290	22,158	0,120		1,01	0,99	1,02					

*Nie* not in equation

Model 1: crude RR

Model 2: adjusted for maternal factors + child factors + health care factors

**p < 0,001

The adjusted intervention risk ratio is displayed in model 2. Consecutive adjustment for maternal, child (Big3 casemix) and health care related factors showed that the planned place of birth had a significant impact on the likelihood of intervention (OR 0.77, [95% CI. 0.75–0.78]) (Table 2, model 2). A similar patern was seen for the planned place unknown group.

### Perinatal mortality

The crude mortality risk ratio was significantly lower for women who planned home birth (RR 0.80 [95% CI 0.71–0.91], *p* < 0.001) compared to those who planned a hospital birth (Table 3, model 1). All maternal and child risk factors, except the presence of a single SGA, showed significant differences in risk ratio in agreement with the expected direction.

**Table 3:** Intrapartum and neonatal death 0–7 days in women who are in primary care at the onset of labour (intention-to-treat-like approach)

	TOTAL(n)	mortality (n)	%	*p*	Model 1			*p*	Model 2			*p*
					Crude RR		95% CI		Adj OR		95% CI	
Intended place of birth				**				**	nie			
Home	402,912	646	0,16%		,80	,71	,91					
Hospital (REF)	219,105	433	0,20%		1,00							
Unknown	57,935	109	0,19%		,96	,77	1,19					
Parity				**				**	nie			
Primiparous	302,489	667	0,22%		1,58	1,40	1,78					
Multiparous (REF)	377,463	521	0,14%		1							
Maternal Age				**				**				**
< 19 years	11,939	44	0,37%		2,43	1,78	3,32		1,79	1,26	2,54	
20–25 years	74,101	146	0,20%		1,24	1,03	1,49		0,99	0,80	1,21	
25–34 years (REF)	478,251	753	0,16%		1				1			
> 35 years	115,661	245	0,21%		1,38	1,19	1,60		1,50	1,28	1,76	
Ethnic background				**				**				**
Dutch (REF)	571,185	953	0,17%		1				1			
Non Dutch	108,767	235	0,22%		1,31	1,13	1,52		1,29	1,09	1,53	
Neighbourhood												**
Privileged neighbourhood (REF)	638,571	1113	0,17%		1				1,00			
Underprivileged neighbourhood	41,381	75	0,18%		1,02	0,80	1,30		0,70	0,53	0,93	
Gestational Age				**				**				**
< 34wk	4621	378	8,18%		87,01	76,10	99,48		27,71	22,58	34,01	
35-36wk	11,780	72	0,61%		5,73	4,43	7,41		2,35	1,75	3,17	
37wk	27,303	61	0,22%		2,12	1,61	2,79		2,04	1,55	2,69	
38-41wk (REF)	626,558	663	0,11%		1				1			
> 41 wk	9690	14	0,14%		1,39	0,80	2,40		1,15	0,66	2,00	
Big4				**								**
SGA	50,681	81	0,16%		0,96	0,76	1,21		2,45	1,92	3,12	
Low apgar	3103	111	3,58%		22,02	17,84	27,18	**	47,56	37,95	59,61	
Congenital abnomality	8296	90	1,08%		5,46	4,28	6,97	**	12,46	9,64	16,10	
Combination Big4	3594	420	11,69%		115,01	101,14	130,77	**	35,96	29,46	43,90	
No Big4	599,445	309	0,05%		1				1			
Time of delivery									nie	nie	nie	
Day 8.00–18.00 (REF)	288,052	499	0,17%		1							
Night 18.00–8.00	391,900	689	0,18%		1,02	0,91	1,16					
Day of delivery									nie	nie	nie	
Week day (REF)	494,662	841	0,17%		1							
Weekend	185,290	347	0,19%		1,12	0,98	1,28					
Intervention*intended place of birth												
Home intervention					2,14	1,80	2,56		1,51	1,25	1,84	
Home no intervention					0,63	0,56	0,71		nie			
Hospital intervention					2,07	1,68	2,55		nie			
Hospital no intervention (REF)					1				1			
Unknown intervention					1,77	1,14	2,76		nie			
Unknown no intervention					1,00	0,80	1,25		nie			

*REF* Referencenie = not in equation

Model 1: crude RR

Model 2: stepwise analysis

* *p* < 0,05 (categories of variables)

** *p* < 0,001(categories of variables)

After adjusting for maternal, child (Big4 casemix) and health care related factors respectively, the stepwise analysis showed that only the interaction term home x intervention was significant (OR 1.51 [95% CI 1.25–1.84]). All other interaction terms (e.g. hospital x intervention) turned out to be non-significant.

Table 4 describes the perinatal mortality rates by intended place of birth and by those with/without intervention for the ten risk groups. The first line describes the noBig3 nulliparous group. The total group consists out of 242,184 women. 116,663 planned their birth at home and received no intervention and 35,179 planned their birth at home and received an intervention. Dividing the intervention rate of the planned home birth group (35,179/35,179 + 116,663) by the intervention rate of the planned hospital group results in the intervention risk ratio (RR = 0.95, column 14). In the planned home group who received no intervention, perinatal mortality occurred in 123 women. This results in a mortality rate of 0.11% in the no intervention group. Dividing the mortality rate of planned home birth by the mortality rate of the planned hospital group results in the mortality risk ratio in the no intervention group (R = (123/116,663)/(78/68,641) = 0.93, column 15).

**Table 4:** Intervention rate (operative vaginal delivery and caesarean section) and mortality (intrapartum and neonatal death 0-7 days post partum) subdivided into riskgroups, parity and place of delivery

				No Intervention group						Intervention Group								
			Total	N		Mortality*		Mortality Rate		N		Mortality*		Mortality Rate		Interven tion rate ratio**	Mortali Ty Rate Ratio No Interven Tion Group***	Mortali Ty Rate Ratio Interven Tion Group****
Pattern			Home + Hospital	Home	Hospital	Home	Hospital	Home	Hospital	Home	Hospital	Home	Hospital	Home	Hospital	Home vs hospital	Home Vs Hospital	Home Vs Hospital
1	NOBIG3	P0	242,184	116,663	68,641	123	78	0,11%	0,11%	35,179	21,701	63	52	0,18%	0,24%	0,95	0,93	0,75
4		P1	310,398	205,324	96,889	127	74	0,06%	0,08%	3582	4603	27	14	0,75%	0,30%	0,37	0,81	2,48
3	SGA	P0	20,071	9186	6799	16	13	0,17%	0,19%	2335	1751	13	5	0,56%	0,29%	0,99	0,91	1,95
8		P1	26,187	16,188	9377	16	7	0,10%	0,07%	281	341	3	1	1,07%	0,29%	0,48	1,32	3,64
3	PREMATURE BIRTH	P0	8752	4366	2582	41	36	0,94%	1,39%	1088	716	6	1	0,55%	0,14%	0,90	0,67	3,95
2		P1	4278	2339c	1592	18	30	0,77%	1,88%	152	195	3	4	1,97%	2,05%	0,53	0,41	0,96
7	CONGENITAL ANOMALY	P0	3785	1511	957	12	6	0,79%	0,63%	815	502	9	5	1,10%	1,00%	1,03	1,27	1,11
4		P1	3740	2300	1253	20	11	0,87%	0,88%	78	109	1	1	1,28%	0,92%	0,39	0,99	1,40
1	COMBINATION BIG3	P0	1134	487	318	34	23	6,98%	7,23%	186	143	10	10	5,38%	6,99%	0,85	0,97	0,77
8		P1	912	487	314	46	29	9,45%	9,24%	54	57	6	3	11,11%	5,26%	0,61	1,02	2,11
	COLUMN		1	2	3	4	5	6	7	8	9	10	11	12	13	14	15	16

*mortality is defined as intrapartum and neonatal death (0-7 days post partum)

**intervention rate home / intervention rate hospital

***perinatal mortality rate home (no intervention) / perinatal mortality rate hospital (no intervention)

****perinatal mortality rate home (intervention) / perinatal mortality rate hospital (intervention)

P0= nulliparous, P1=multiparous

P0= nulliparous, P1=multiparous

Relatively high mortality rates were seen within in the primiparous with a Big3 combination (6.8%, home and hospital data combined, data not shown) and multiparous women (9.2%, home and hospital data combined, data not shown). The intervention rate was lower for women who planned a home birth, except for congenital anomalies in primiparous women (RR =1.03).

Grouping the intervention risk ratios into various risk groups, we observed the patterns as depicted in Table 4.

The primiparous NoBig3 (pattern 1) accounts for 39% of all deliveries, and the multiparous NoBig3 group (pattern 4) accounts for 50% of all deliveries, the remaining risk groups for 11%.

## Discussion

### Main findings

In this study the adjusted intervention rate in multiparous women with a planned home birth was lower compared to women with a planned hospital birth. We cannot provide a judgement on the presence of over- and undertreatment in general, but in some specific risk groups undertreatment at home may be present. Overtreatment in the hospital might be present in the noBig3 multiparous women. However, the benefit of substantially fewer interventions in the planned home group seems to be counterbalanced by substantially increased mortality if intervention occurs.

The most important observation seems that Big3 pregnancies at home show a mortality disadvantage, suggesting undertreatment through delayed timing of intervention or a too high threshold for intervention. One important source of delay is travel time from home to the hospital. Amelink et al. found that 0.4% of all low risk pregnancies need urgent referral. In the Netherlands, average time to the nearest hospital is about 13 min (ranging from 0 to 60 min). They concluded that the net travel time from home to hospital of 20 min or more by car is associated with an increased risk of mortality and adverse outcomes in term women [29]. Moreover, Ravelli et al. found that delivery at 37 weeks or 41 weeks of gestation in combination with travelling time increased the risk of mortality even further [30]. A second source of delay is the delay of the referral decision as suggested by Evers [22]. They observed a more than 3.5-fold higher perinatal death rate in infants of women who were referred from primary to secondary care during labor compared with infants of women who started labor in secondary care.

### Strengths and limitations

A strength of this study was the size and completeness of the study population, covering the complete Dutch births from 2000 to 2007. The amount of missing data was negligible and mortality data have been shown to be complete. Annual trends in the studied relations were absent, except for a minimal gradual decrease in total perinatal mortality [15].

Our casemix adjustment turned out to be essential. We previously showed that, within the low risk group of midwife led deliveries, unequal prevalence of Big4 conditions is present in planned home versus hospital births. This suggests an unequal risk load at the onset of childbirth since, either due to self-selection or due to the midwife’s proposal, the healthiest and most affluent women are more likely to undertake a home birth. Without adjusting for this, one introduces confounding by indication bias [26].

Another strength was the inclusion of women who according to delivery guidelines should have been referred to the obstetrician before the onset of labor, but were not recognized as such in the antenatal phase as health care performance during labor should include the performance of care during the preceding antenatal phase in terms of distinguishing between low and high risk [26].

Several limitations merit discussion. Firstly, an RCT would theoretically be the superior design to address our research question. However the only RCT on home versus hospital birth resulted in low participation rates and selective participation [31]. Treatment groups composed on the basis of women’s preference for setting is likely to affect outcome, producing biased estimates of setting effects. Hence an RCT design is unfeasible within our obstetric system [31–33]. As next-best option, we applied casemix adjustment to the extent the data permitted. We assume Big4 adjustment corrects for the major risk differences at the time of birth. It does not adjust for unmeasured risk differences associated with the remaining 15% of perinatal deaths which are unrelated to the Big4. If we assume these risks follow the same pattern associated with the ‘healthy home birth’, our adjustment is still conservative. Secondly, long term child outcomes in terms of e.g. psychomotor development and behavioral function are needed to confirm whether they parallel the mortality pattern. Thirdly, few data are available on the precise clinical assessment leading to referral or intervention which would allow for better judgement on setting-dependent over- or undertreatment in our analysis. Lastly, our study is also limited in that only intervention rate and mortality are used as outcome indicators, ignoring the mother’s experience. However, studies addressing the trade-off between intervention consequences for the mother (e.g. caesarean section) versus safety of the child clearly indicate that even small advantages to the child’s outcome outweigh the consequences of an intervention [34].

Our results appear compatible with most of the few available reports on this issue. Previous studies on planned home births attended by registered community midwives confirm the lower risk of receiving an intervention and suggest equal mortality [2–12]. However these studies are limited by lack of applying complete casemix adjustment, thereby suggesting risk equivalence of home and hospital groups [2, 3, 6–12], afterwards exclusion of unplanned and unsuitable home births from analysis. [2, 6–8, 10, 11], voluntary submission of data [2, 6–8, 10, 11], or lack of statistical power [2, 3, 6, 8, 9, 11]. These limitations generally tend to benefit outcome in favor of home birth. Sofar, none of these studies has performed a case fatality analysis based on predefined risk groups. The birthplace in England Collaborative Group concluded from their subgroup analysis fewer interventions in planned homebirths compared to planned hospital births, associated with an increased incidence of the adverse perinatal outcome for nulliparous women. For multiparous women, there were no significant differences in adverse perinatal outcome [4, 20].

Women with an unknown place of birth tend to have similar characteristics as women planning their birth in the hospital. More detailed research should be done to this group.

## Conclusion

The planned place of birth impacts the intervention rate in an assumed low risk population. In planned home births especially multiparous women showed universally lower intervention rates. However, the benefit of substantially fewer interventions in the planned home group seems to be counterbalanced by substantially increased mortality if intervention occurs. If risk selection can be improved both in terms of detection and timely referral, multiparous women could experience benefits from the non-medical setting. A perinatal mortality disadvantage of the home setting can be observed in undetected risk groups. More research should be done on the precise clinical assessment leading to referral or intervention which would allow for better judgement on setting-dependent over- or undertreatment. This study helps policy makers to gain a more balanced view in the discussion regarding advantages and disadvantages of the different places of birth.

## Abbreviations

PRN: Netherlands Perinatal Registry
SGA: Small for Gestational Age
